# Liquid Nitrogen-Based Cryoablation in In Vivo Porcine Tissue: A Pilot Study

**DOI:** 10.31557/APJCP.2020.21.10.3069

**Published:** 2020-10

**Authors:** Doyoung Chang, Prasoon Mohan, Ayush Amin, Monica Garcia-Buitrago, Jose Rodriguez, Robert Peaden

**Affiliations:** *University of Miami, Miller School of Medicine, 1600 NW 10th Ave #1140, Miami, FL 33136, USA. *

**Keywords:** Cryoablation, cryotherapy, liver cancer, kidney cancer, minimal invasive surgery

## Abstract

**Introduction:**

Liquid nitrogen-based cryoablation induces freezing evenly throughout the probe tip surface, resulting in larger ablation volumes and faster treatment times. The purpose of this preliminary investigation is to determine the efficacy of the liquid nitrogen-based Visica2 Cryoablation System (Sanarus Technologies, Pleasanton, CA) in in vivo porcine kidney, liver, and fibro-fatty tissue.

**Methods:**

Ablations were performed under ultrasound guidance in 4 Yorkshire pigs. The target lesion cross-section width (W) and depth (D) were 1 cm for liver (n=8), kidney (n=4), and head-neck (n=5) and 2 cm for kidney (n=4). Expected axial length (L) of the resulting lesion is approximately 4 cm. After three-day survival, the ablated tissue was harvested and histologically analysed. The mean width and depth were compared with the target diameter using a one-sample t-test.

**Results:**

All animals survived the procedure. For the 1 cm target, mean dimensions (L x W x D) were 3.8±1.5 x 1.7±0.3 x 1.7±0.7 for liver, 3.0±0.5 x 2.0±0.4 x 1.7±0.6 for kidney, and 3.3±0.8 x 1.8±0.4 x 1.8±0.4 for head-neck. Mean width and depth were significantly greater than desired dimension. For the 2 cm target, mean dimensions were 3.2±0.5 x 3.1±0.8 x 1.9±0.7. Mean width and depth were not significantly different to desired target.

**Conclusion:**

Our preliminary results show that the Visica2 liquid nitrogen-based cryoablation system can efficiently and reproducibly create ablation volumes in liver, kidney, and fibro-fatty tissue within 4 minutes and 12 minutes for 1cm and 2cm targeted diameters, respectively. Further investigation is necessary to determine the optimal freeze-thaw-freeze protocol for larger ablation volumes.

## Introduction

Cryoablation systems using argon gas as the cryogen have been used effectively for the treatment of small tumors in liver, kidney, prostate, breast and lungs (Bahn et al., 2002; Seifert and Junginger, 2004; Gill et al., 2005; Kawamura et al., 2006; Nakatsuka et al., 2010; Ito et al., 2012; Poplack et al., 2015). Two widely used systems in the United States are the Endocare system (Healthronics, Austin, Texas) and Galil Medical’s cryoablation system (Galil Medical, Arden Hills, Minnesota) (Erinjeri and Clark, 2010). In these systems, one or multiple probes are inserted into the tumor under ultrasound or computed tomography guidance, and then compressed argon gas is perfused into the probes. Consequently, there is sudden expansion of the argon gas within the distal tip of the probe, which makes the gas, and thus the probe, extremely cold (−140 to −150°C)(van Leeuwen et al., 2014; Shrivastava, 2018). Heat is then conducted toward the probe from the surrounding tissue, creating an ice ball that encompasses the tumor. Probe thawing is achieved by perfusing Helium gas, which gets warm during sudden expansion, by altering the argon gas pressure, or by pulsing the argon gas emission (Edwards et al., 2004).

The Visica 2 Cryoablation Treatment System (Sanarus Technologies, Pleasanton, CA), uses low-pressure liquid nitrogen rather than argon as the cryogen and a resistance heater for thawing. Liquid nitrogen cryogen systems achieve rapid freezing at the probe tip (−160 to −170 °C), which may also allow shorter treatment times (Golatta et al., 2015) and larger treatment volumes(Hewitt et al., 1997a). Also, unlike argon which requires a room with specific safety mechanisms that limits its use, liquid nitrogen can be used in a conventional operating room since it is stored within a non-pressurized container (Nomori et al., 2017a).

Currently, the Visica 2 is indicated for use in ablation of benign and malignant breast tumors (Simmons et al., 2016). Preliminary studies done by Nomori et al demonstrated the treatment potential of cryoablation using liquid nitrogen in ex vivo porcine lung tissues(Nomori et al., 2017b), but no study has yet been done to evaluate its performance in an in vivo liver or kidney model. Thus, this preliminary study aims to explore the feasibility of using Visica 2 in treating solid tumors in liver, kidney, and fibro-fatty tissue through an in vivo porcine experiment. The primary goal of this study was to compare the anticipated and observed ablation dimensions, with secondary measures including the determining of the safety of the device. 

## Materials and Methods

All animal experiments performed in this study were performed under the Institutional Animal Care and Use Committee (IACUC) approved protocol. Percutaneous cryoablation was performed using the Visica 2 Liquid-Nitrogen Cryoablation System on four female Yorkshire pigs using a 12 gauge cryoprobe. Each animal weighed approximately 100 lbs. 

The Visica 2 Cryoablation Treatment System consists of a generator with a touch screen user interface and an ICE 10-12 gauge needle probe. The probes have a 4.0 cm freezing tip and an outer diameter of 3.4 mm. Liquid nitrogen is infused into and circulated out of the probes, causing a rapid cooling within the surrounding tissue. The probes consist of a surgical stainless steel needle containing an electrical warming component for thawing and an integrated thermocouple for internal temperature feedback.

Cryoablation was performed using a double freeze-thaw-freeze cycle. Predetermined manufacturer freeze-thaw cycle phase periods were used based on the cross-sectional ablation diameter. The duration of the freeze-thaw-freeze cycle was 1-2-1 and 2-8-2 minutes for target diameters of 1 cm and 2 cm, respectively. Target ablation size was determined based on the size and anatomy of the organ. Accordingly, 1cm targeted diameter ablation was performed in the liver (n=8), upper pole of the kidney (n=4), and fibro-fatty tissue in the head-neck region (n=5). In the lower pole of the kidney (n=4), 2cm targeted diameter ablations were performed.

**Figure 1:**
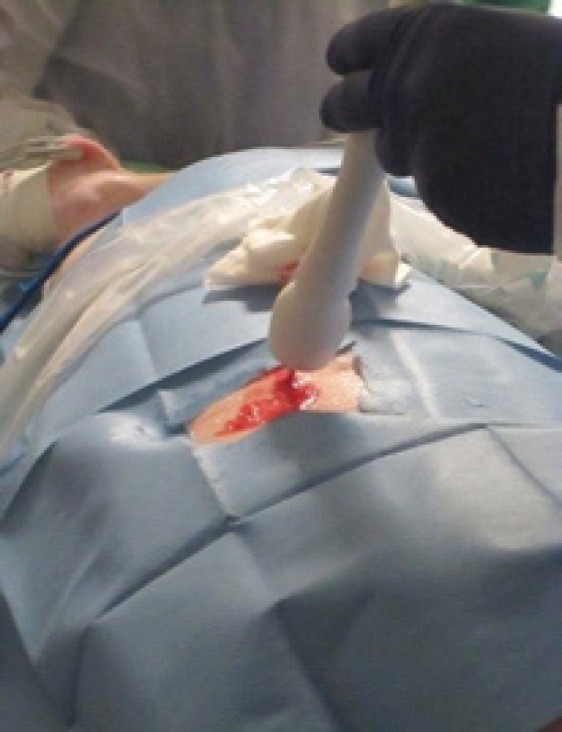
Sanarus ICE Probe Inserted Percutaneously into an Animal under Ultrasound Guidance

A water test, in which an ablation was performed within a small volume of water, was done prior to percutaneous ablations to ensure that the probe was functioning properly. The animals were given anesthesia during the procedure and analgesics afterwards. After the animals were anesthetized, the needle was inserted percutaneously under ultrasound guidance (Figure 1). 

Ablations were first performed in the right and left hepatic lobe of each animal. The procedure was then repeated for the upper and lower pole of the right kidney of each animal. Ablations were only performed on one kidney per animal in order to survive the animal. An identical procedure was then repeated in the fibro-fatty tissue within the head-neck region. Saline was injected between the skin and the fibro-fatty tissue in two cases and between the tissue and muscle in one case to puff up the tissue volume, due to the minimal amount of fibro-fatty tissue within the head-neck region.

After all ablations were completed, the animals were given analgesics and survived for 3 days. On the third day of survival, each animal was euthanized, and its ablated organs were harvested for histopathological analysis. Each harvested organ was serially sectioned at 0.5 cm intervals. Composite sections from the organ hilum to the surface or from the deep areas to the skin were obtained and place on cassettes for histological analysis. The tissues were processed, embedded in paraffin, cut at 3 micron thickness, and stained with Hematoxylin and Eosin (HandE). Mirror composite sections were also obtained, frozen in liquid nitrogen and sliced into cryostat sections using a cryostat refrigerated microtome and stained with Nicotinamide Adenine Dinucleotide (NADH) diaphorase.

The ablation volume cross-section width (W), depth (D) and axial length (L) were measured and compared with the target values(Table 1) (Brace et al., 2009). All continuous variables were expressed as mean**±**SD. Statistical analysis was performed using SPSS-24 (IBM^©^ Corp., Armonk, NY). In all analyses, a one-way t-test was used and the p value of <0.05 was considered statistically significant different.

## Results

*Dimensions of Zones of Ablation*

**Figure 2:**
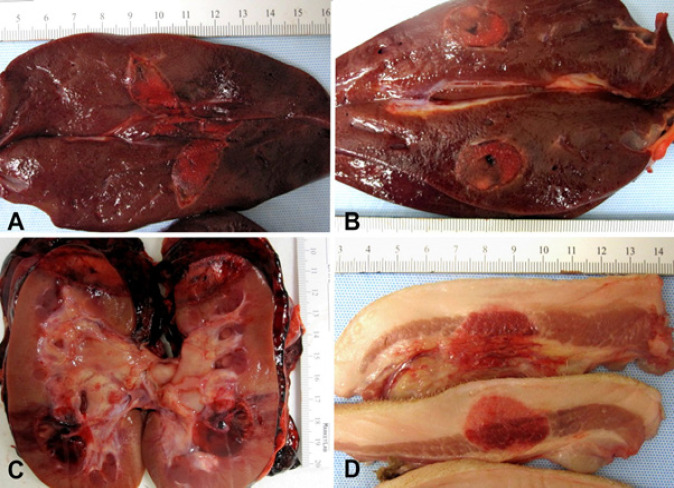
A-B. Right and left liver lobes with well demarcated ablation areas. C. Kidney upper and lower pole ablated areas with marked hemorrhage in perinephric fat. D. Head and neck fibro-fatty and muscular tissue with a well circumscribed ablated area

All animals survived the procedure. The ablation areas were well demarcated as can be seen in Figure 2. Ablations with a targeted diameter of 1 cm was performed in the liver, upper pole of the kidney, and fibro-fatty tissue in the head-neck region. For the liver (n=8), mean dimensions (L x W x D) of the ablation volume were 3.8±1.5 x 1.7±0.3 x 1.7±0.7. For the upper lobe of the kidney (n=4), mean dimensions were 3.0±0.5 x 2.0±0.4 x 1.7±0.6. For fibro-fatty tissue in the head-neck region (n=5), mean dimensions were 3.3±0.8 x 1.8±0.4 x 1.8±0.4. Both the overall average width and depth were significantly greater than the targeted 1cm diameter (p<0.05). Results are summarized in Table 1. 

For 2 cm target ablations in the lower pole of the kidney (n=4), mean dimensions were 3.2±0.5 x 3.1±0.8 x 1.9±0.7. While the achieved width were statistically significantly greater than their corresponding targets, depth were not statistically significantly larger than targeted values. 

*Histological and Macroscopical Analysis*

Macroscopic examination of the harvested organs showed well circumscribed ablated areas surrounded by variable amount of hemorrhage that ranged from minimal in the soft tissue to more evident in the kidneys. Gross pictures of ablated organs are shown in Figure 2.

**Figure 3:**
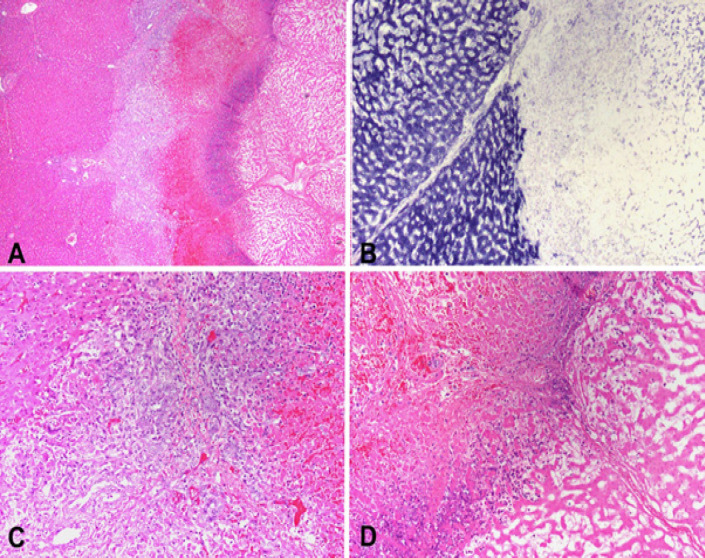
A. Periphery of an ablated zone demonstrating from left to right: viable liver, inflammatory response, hemorrhagic necrosis, nuclear dust and necrotic ablated liver (H and E stain). B. NADH staining showing viable liver on the left and necrotic ablated liver on the right. C. High power of viable liver on the left followed by bile duct reaction, lymphocytes, plasma cells, eosinophils, and hemorrhagic necrosis (H and E stain). D. High power showing a rim of nuclear dust and coagulative necrosis on the right (H and E stain)

All the organs demonstrated overall architectural preservation of the ablated areas on microscopic examination with HandE staining. The ablated areas revealed coagulative necrosis, featuring increased eosinophilia, indistinct cellular borders, and ghost nuclei. The necrosis was surrounded by a rim of nuclear dust, hemorrhagic necrosis, and a variable degree of lymphoplasmacytic inflammatory infiltrate with reactive myofibroblasts. The NADH staining was negative within the ablation zones, indicating cellular death. Ablation zones in the liver (n=8) showed a well-demarcated area of necrosis surrounded nuclear dust rim, followed by hemorrhagic necrosis and an inflammatory response, which included lymphocytes, plasma cells, and myofibroblasts, and was accompanied by reactive bile ductular reaction (Figure 3). 

**Figure 4:**
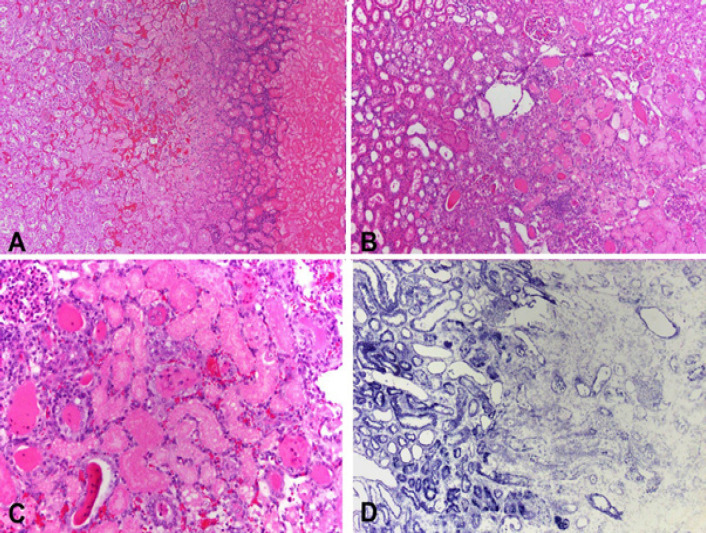
A. Low power section showing from left to right viable renal parenchyma followed by a congested area with tubular necrosis, a rim of nuclear dust and coagulative necrosis (H and E stain). B. Scant inflammation between viable renal parenchyma (left) and necrosis (right) (H and E stain). C. Necrotic proximal renal tubules adjacent to distal tubules showing luminal proteinaceous material (H and E stain). D. NADH staining demarcating viable (left) from necrotic (right) parenchyma

Ablation zones in the kidney (n=8) were surrounded by a rim of nuclear dust, congestion and necrotic proximal renal tubules admixed with distal renal tubules containing intraluminal proteinaceous material. The inflammatory infiltrate was scant. As observed upon gross examination, a hematoma in the perirenal fat was present (Figure 4).

**Figure 5:**
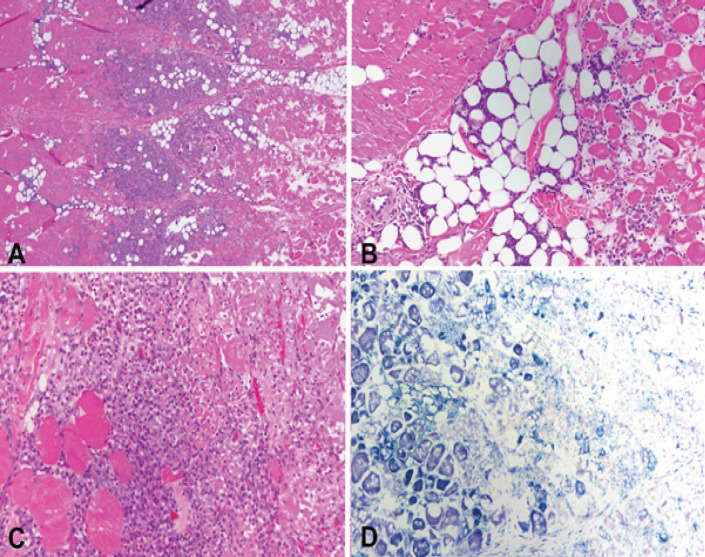
A. Head and neck soft tissue and muscle showing viable tissue (left), marked inflammatory reaction (middle) and necrotic tissue (right) (H and E stain). B. Organized viable (left) and necrotic (right) muscle fibers (H and E stain). C. Marked inflammatory infiltrate comprised of neutrophils, plasma cells and myofibroblasts, adjacent to necrotic muscle bundles (right lower) (H and E stain). D. NADH staining showing positive viable muscle fibers (left) and negative necrotic tissue (right)

Necrotic skeletal muscle surrounded by a lymphoplasmacytic and neutrophilic inflammatory infiltrate with a myofibroblastic reaction and extending to adjacent adipose tissue was observed in the ablated areas of the head and neck fibro-fatty tissue (Figure 5).

**Table 1:** The Anticipated and Actual Measured Dimensions of the Ablation Dimensions (cm)

Targeted			Measured		
Diameter (cm)	Organ	Sample Size	Length (cm)	Width (cm)	Depth (cm)
1	Liver	8	3.8±1.5	1.7±0.3	1.7±0.7
	Kidney	4	3.0±0.5	2.0±0.4	1.7±0.6
	Fibro-fatty Tissue	5	3.3±0.8	1.8±0.4	1.8±0.4
2	Kidney	4	3.2±0.5	3.1±0.8*	1.9±0.7*

*, not statistically significant

## Discussion

Cryoablation utilizes temperatures below -20^o^C to destroy tumors by fast freezing of the tissue followed by slow thawing, and with a second freeze-thaw cycle often conducted in order to achieve maximum destructive effect and destroy any potential residual tumor cells in the targeted lesion (Jacob et al., 1985; Gage and Baust, 1998; Li et al., 2015; Wu et al., 2015). Each phase results in a different type of cellular injury. In the freezing phase, ice crystals are formed by freezing intracellular and extracellular water, leading to cell necrosis and cell lysis at the center of the targeted lesion. In the thawing phase, cell death is achieved by apoptosis, where endothelial damage in the treatment zone can lead to peripheral ischemia of the lesion, and necrosis of the treated cells can later release antigens that stimulate an immune response (Gage and Baust, 1998). Different temperatures and freeze-thaw cycles can be selected depending on the type of tissue and its sensitivity to cold, and thus requires experimental evaluation to further optimize the operation parameters. 

The visceral organs in this study have traditionally been treated via percutaneous cryoablation with argon gas, which has been demonstrated to be effective in the treatment of malignant tumors of the liver, prostate, kidney, and lung (Bahn et al., 2002; Seifert and Junginger, 2004; Gill et al., 2005; Wang et al., 2005; Kawamura et al., 2006; Nakatsuka et al., 2010; Ito et al., 2012). Argon gas cryoablation relies on the Joule-Thompson effect, where pressurized argon gas is delivered to the tip of the cryoprobe and expanded through a minute pore. This in turn, rapidly reduces the temperature at the needle tip and forms ice balls used for cryoablation (Tay et al., 2016). While it is capable of causing freezing levels of up to −140°C in tissue within 60 seconds(van Leeuwen et al., 2014; Lv et al., 2016), the use of pressurized argon gas requires a room with specific safety mechanisms which limits its use (Nomori et al., 2017a).

Liquid nitrogen on the other hand, is infused from a simple dewar (-196°C) into the probe, allowing it to be performed in a conventional operating room (Nomori et al., 2017a). In a typical liquid nitrogen- based cryoablation system, liquid nitrogen changes its phase and turns into gas at the tip of the probe, which is relatively warmer (Hewitt et al., 1997b). This layer of gas formed between the liquid nitrogen and the probe insulates the probe from the liquid nitrogen, resulting in the temperature of the probe tip to be around -160C (Hewitt et al., 1997b). Another limitation of nitrogen cryoablation is vapor lock. When the cryogen is expelled from the needle, it quickly transforms from liquid to gas, and the rapid gas expansion can create a backward pressure and hinder the forward flow of the liquid nitrogen through the needle(Erinjeri and Clark, 2010). The Visica 2 system used in this study uses low pressure liquid nitrogen and is able to eliminate vapor lock by removing the transition phase of liquid to gas. 

The use of liquid nitrogen has been more extensively studied in the treatment of breast fibroadenoma, gastrointestinal tract diseases such as Barrett esophagus, early esophageal cancer, and human papillomavirus (HPV) warts and skin tumors(Gosain et al., 2013; Hahn et al., 2013; Kasuya et al., 2015; Yang et al., 2015). Conventionally, Visica 2 system has been used in the treatment of breast fibroadenomas and gynecological neoplasia involving the female genitalia and has been demonstrated to be effective in 92% of targeted lesions and to have 100% ablation in tumors <1.0cm (Simmons et al., 2016). 

In this study, we attempted to further investigate the feasibility of using liquid nitrogen-based cryoablation and evaluate its efficacy in the treatment of visceral organs and fibro-fatty tissue. A previous study has demonstrated the feasibility of liquid nitrogen-based cryoablation in an ex vivo porcine lung tissue(Nomori et al., 2017b), but it has yet been evaluated in the liver, kidney or fibro-fatty tissue in an in vivo porcine model. The results of our preliminary experiment indicate that liquid nitrogen-based cryoablation may be feasible in achieving sufficient ablation volumes to treat small tumors in the liver and kidney. While the current results were limited in testing larger target volumes due to the size of the organs, results suggest that larger ablation volumes may be achieved with longer freeze-saw cycles. Also, shorter treatment times may be possible for smaller lesions with 1cm target ablation diameter. Results suggest the need of further investigation to determine optimal freeze-thaw cycle parameters in larger sample sizes. 

Ideally, the width and depth of the ablation volume should have been of similar dimensions in order to create a circular cross section and reduce collateral damage to the adjacent structures. However, ablation size may differ even with identical settings, due to tissue of different compositions responding differently to a certain temperature, and convective warming of the blood flow to a tissue area(Wood et al., 2007; Khairy and Dubuc, 2010)

Complications in this study involved extension of the ablation zone into the skeletal muscle in two fibro-fatty tissue ablations. Hemorrhage was observed between the capsule surface and Gerota’s fascia in all renal cases. We postulate that the hemorrhage was due to the relatively large 10G and 12G ablation probes which were specifically designed for breast ablation. This suggests the need of a smaller probe for ablation in the liver or kidney. It should also be mentioned that the disadvantage of the current Visica 2 system is the lack of the ability to use multiple probes with each device, compared to argon gas cryoablation systems (Arnott, 1851; Nomori et al., 2017b).

One of the this pilot trial is the lack of comparative studies. While the results cannot be directly compared to the previous study in the lung tissue using liquid nitrogen, the results somewhat agree that larger ablation volumes may be achieved using liquid nitrogen compared to argon gas (Nomori et al., 2017b). While all ablations have reached or exceeded its planned volume, the lack of a sufficient sample size to determine accurate freeze-thaw phase period settings to achieve a given target size is another limitation. Nonetheless, results are promising and suggest the need of further investigation to determine accurate settings for different organs and tissue types. Additionally, animals were only allowed to survive for 3 days due to limitations in resources, and a longer survival may be necessary to adequately determine the safety of the Visica 2 system in the ablation of these visceral organs and tissue. 

In conclusion, liquid nitrogen cryotherapy with the Visica 2 system may have a potential role in the treatment of liver, kidney, and fibro-fatty tissue. Ablation volumes up to 1cm and 2cm targeted diameters were achieved within 4 minutes and 12 minutes, respectively. Further investigation is necessary to determine the optimal freeze-thaw-freeze protocol for larger ablation volumes and ablations in different tissue types.
